# 790例初诊急性早幼粒细胞白血病患者临床及遗传学特征的单中心回顾性研究

**DOI:** 10.3760/cma.j.issn.0253-2727.2022.04.012

**Published:** 2022-04

**Authors:** 娩增 杨, 乐 李, 辉 魏, 兵城 刘, 凯奇 刘, 大鹏 李, 磊 张, 仁池 杨, 营昌 秘, 建祥 王, 迎 王

**Affiliations:** 中国医学科学院血液病医院（中国医学科学院血液学研究所），实验血液学国家重点实验室，国家血液系统疾病临床医学研究中心，细胞生态海河实验室，天津 300020 State Key Laboratory of Experimental Hematology, National Clinical Research Center for Blood Diseases, Haihe Laboratory of Cell Ecosystem, Institute of Hematology & Blood Diseases Hospital, Chinese Academy of Medical Sciences & Peking Union Medical College, Tianjin 300020, China

**Keywords:** 白血病，早幼粒细胞，急性, 细胞遗传学, 突变基因, Leukemia, promyelocytic, acute, Cytogenetics, Mutated gene

## Abstract

**目的:**

探讨初诊急性早幼粒细胞白血病（APL）患者的临床及遗传学特征。

**方法:**

纳入2004年2月至2020年6月期间于中国医学科学院血液病医院就诊的15岁及以上初诊APL患者，对其临床及实验室特征进行回顾性分析。

**结果:**

共收集790例APL患者，男女比例为1.22∶1，中位年龄41（15～76）岁，以20～59岁为主。中低危组患者632例（80％），高危组患者158例（20％）。4.8％的初诊患者合并银屑病。初诊患者的WBC、PLT、HGB水平分别为2.3（0.1～176.1）×10^9^/L、29.5（2.0～1220.8）×10^9^/L、89（15～169）g/L。PML-RARα亚型以 L型最常见，占58％。初诊患者很少出现APTT延长（10.3％）和肌酐>14 mg/L（1％）。对715例患者进行核型分析，155例（21.7％）为t（15;17）伴附加染色体异常，其中以+8（5.5％）最常见。复杂核型见于64例（9.0％）患者。对178例患者进行二代测序检测，共检出113个突变基因，发生率>1％的突变基因有75个，以FLT3（44.9％）最常见；FLT3-ITD见于20.8％的患者。

**结论:**

本中心APL患者以中青年为主，男女比例未见明显差异。危险分层以中低危患者为主。少部分患者伴附加的染色体异常，以+8最常见。PML-RARα 亚型以L型最常见。APL突变谱以FLT3最常见。

## Abstract (en)

**Objective:**

To retrospectively analyze the data of Chinese patients with newly diagnosed acute promyelocytic leukemia（APL）to preliminarily discuss the clinical and cytogenetic characteristics.

**Methods:**

From February 2004 to June 2020, patients with newly diagnosed APL aged ≥ 15 years who were admitted to the Institute of Hematology & Blood Diseases Hospital, Chinese Academy of Medical Science & Peking Union Medical College were chosen. Clinical and laboratory features were retrospectively analyzed.

**Results:**

A total of 790 cases were included, with a male to female ratio of 1.22. The median age of the patients was 41（15–76）years. Patients aged between 20 and 59 predominated, with 632 patients（80％）of 790 patients classified as low and intermediate risk and 158 patients（20％）of 790 patients classified as high risk. The white blood cell, platelet, and hemoglobin levels at diagnosis were 2.3（0.1–176.1）×10^9^/L, 29.5（2.0–1220.8）×10^9^/L, and 89（15–169）g/L, respectively, and 4.8％ of patients were complicated with psoriasis. The long-form type of PML-RARα was most commonly seen in APL, accounting for 58％. Both APTT extension（10.3％）and creatinine >14 mg/L（1％）are rarely seen in patients at diagnosis. Cytogenetics was performed in 715 patients with newly diagnosed APL. t（15;17）with additional chromosomal abnormalities were found in 155 patients, accounting for 21.7％; among which, +8 was most frequently seen. A complex karyotype was found in 64（9.0％）patients. Next-generation sequencing was performed in 178 patients, and 113 mutated genes were discovered; 75 genes had an incidence rate>1％. FLT3 was the most frequently seen, which accounted for 44.9％, and 20.8％ of the 178 patients present with FLT3-ITD.

**Conclusion:**

Patients aged 20–59 years are the most common group with newly diagnosed APL. No obvious difference was found in the ratio of males to females. In terms of risk stratification, patients divided into low and intermediate risk predominate. t（15;17）with additional chromosomal abnormalities accounted for 21％ of 715 patients, in which +8 was most commonly seen. The long-form subtype was most frequently seen in PML-RARα-positive patients, and FLT3 was most commonly seen in the mutation spectrum of APL.

急性早幼粒细胞白血病（APL）为血液系统恶性疾病，占成人急性髓系白血病（AML）的5％～30％[1]，年发病率为0.112～0.318/10万[2]–[5]，t（15; 17）（q24; q21）为其典型遗传学改变，形成特征性PML-RARα融合基因。2016年WHO将该病更名为APL伴PML-RARα[6]，以此来区分隐匿性遗传学异常和变异型APL。近年来随着二代测序等基因检测技术的开展，APL的突变谱在不断扩大，并由此发现了越来越多的重现性突变基因，为全面深入认识该疾病及精准治疗提供了依据。我们回顾性分析了我院790例初诊APL患者的临床及遗传学特征，以进一步了解中国人群该疾病的情况。

## 对象与方法

1. 研究对象：2004年2月至2020年6月于我院就诊的15岁及以上初诊APL患者共790例。诊断符合WHO 造血及淋巴组织分类标准。本研究符合赫尔辛基宣言，获得所有受试者书面同意。

2. 免疫分型：检测标本为初诊患者肝素抗凝的骨髓。采用CD45/SSC 设门法，标记的抗体包括CD34、CD45、HLA-DR、CD11b、CD13、CD38、CD117、CD16、CD15、CD33、CD123、CD56、CD7、CD64、CD14、CD71、CD4、CD19、CD10、cCD3、mCD3、cCD79a、CD2、CD5、MPO和TdT。采用Beckman Coulter FC-500和BD FACS Canto Ⅱ流式细胞仪进行检测。采用单克隆抗体四色或八色直接荧光标记法，每个样本获取10 000 个细胞。CD45/SCC 设门后的白血病细胞群膜表面抗原>20％判断为阳性[7]，胞内抗原>10％判断为阳性[8]。

3. 细胞遗传学：染色体标本取自骨髓，肝素抗凝。经常规24 h短期培养，收获后制片，R显带或R+G显带。依据《人类细胞遗传学国际命名体制（ISCN2013）》描述克隆性染色体畸变。如果未见到足够的t（15;17）细胞分裂象，但染色体荧光原位杂交和（或）融合基因检测证实PML-RARα阳性，允许纳入数据分析范围。如果细胞正常核型和染色体畸变同时存在，将其纳入染色体畸变。不存在t（15;17）和正常核型，但存在克隆性染色体畸变的纳入其他染色体数目和结构异常。附加染色体异常（ACA）定义为除t（15;17）［不限于孤立的t（15;17）］外还伴其他的克隆性染色体畸变。复杂核型定义为3个或更多的克隆性染色体畸变，包括t（15;17）。

4. 分子生物学：PML-RARα、TP53基因缺失检测采用荧光原位杂交方法。分别选择双色双融合标记PML-RARα探针、双色标记TP53探针进行变性及杂交。每个标本分析500个间期细胞。BCR-ABL、RUNX1-RUNX1T1、PML-RARα、PLZF-RARα、NPM-RARα、STAT5b-RARα、MLL-ELL和NUP98-PMX1等融合基因的检测采用实时定量PCR法。本组有178例患者采用二代测序方法检测与血液系统疾病密切相关基因的蛋白质编码区域的点突变和短片段插入/缺失突变。

5. 统计学处理：符合正态分布的连续变量组间比较采用独立样本*t*检验；不符合正态分布采用非参数检验（Mann-Whitney *U*检验）；分类变量比较采用*χ*^2^检验及Fisher精确检验。*P*值为双侧，*P*<0.05为差异有统计学意义。使用SPSS 23.0 软件进行统计学分析。

## 结果

1. 患者的临床和实验室特征：2004年2月至2020年6月于我院就诊的15岁及以上初诊APL患者共790例。其中男434例（55％），女356例（45％），男女比例为1.22∶1。中位年龄41（15～76）岁，以20～59岁患者为主。发病时WBC、PLT、HGB分别为2.3（0.1～176.1）×10^9^/L、29.5（2.0～1220.8）×10^9^/L、89（15～169）g/L。4.8％的初诊患者合并银屑病。APL患者以PML-RARα L型最常见，占58％。进一步按照Sanz危险分层标准[9]将患者分为中低危组和高危组。体重分层按照WHO亚洲标准[10]。患者的临床特征见表1，实验室特征见表2。中低危组患者632例（80％），高危组患者158例（20％），高危组的中位年龄小于中低危组。高危组中位凝血酶原时间、中位乳酸脱氢酶水平高于中低危组，而中位纤维蛋白原水平低于中低危组。此外，初诊患者很少出现APTT延长（10％）和肌酐>14 mg/L（1％）。就PML-RARα亚型而言，中低危组以L型为主，占61％；而高危组L型和S型比例相似，分别为47％和45％。790例患者中451例进行了FLT3突变检测，采用二代测序方法对其中178例患者进行基因突变检测，其余均为一代测序结果。FLT3-ITD突变与WBC>10×10^9^/L（*P*＝1.000）及免疫表型（CD2、CD56、CD11b）与PML-RARα亚型（L、S、V）（*P*值均为1.000）之间均未见相关性。

**表1:** 790初诊急性早幼粒细胞白血病患者的临床特征［例（％）］

特征	所有患者（790例）	中低危组（632例）	高危组（158例）	*P*值
性别				0.946
男	434（55）	341（54）	93（59）	
女	356（45）	291（46）	65（41）	
年龄（岁）				<0.001
15～19	48（6）	28（4）	20（13）	
20～29	138（18）	105（17）	33（21）	
30～39	176（22）	139（22）	37（23）	
40～49	217（27）	182（29）	35（22）	
50～59	137（17）	116（18）	21（13）	
60～69	67（9）	56（9）	11（7）	
70～79	7（1）	6（1）	1（1）	
ECOG评分				0.955
0～1	203（84）	164（85）	39（81）	
2～4	38（16）	29（15）	9（19）	
BMI（kg/m^2^）				0.286
≤22.9	176（32）	141（32）	35（31）	
23.0～24.9	111（20）	99（23）	12（11）	
25.0～29.9	202（37）	148（34）	54（48）	
≥30.0	61（11）	50（11）	11（10）	
吸烟				0.847
有	193（27）	149（27）	44（30）	
无	512（73）	411（73）	101（70）	
饮酒				0.127
有	157（22）	122（22）	35（25）	
无	543（78）	436（78）	107（75）	
银屑病				1.000
有	38（14）	32（15）	6（11）	
无	225（86）	177（85）	48（89）	

注：ECOG：美国东部肿瘤协作组；BMI：体重指数

**表2:** 790例初诊急性早幼粒细胞白血病患者的实验室特征

特征	所有患者（790例）	中低危组（632例）	高危组（158例）	*P*值
WBC［×10^9^/L，*M*（范围）］		1.7（0.1～10.0）	23.8（10.0～176.1）	
PLT［×10^9^/L，*M*（范围）］		31.0（2.0～1220.8）	26.0（4.0～294.0）	0.008
PLT［例（％）］				
≤40 ×10^9^/L	504（64）	384（61）	120（76）	
（41～100）×10^9^/L	211（27）	177（28）	34（22）	
>100×10^9^/L	75（9）	71（11）	4（2）	
HGB［g/L，*M*（范围）］		89（24～169）	87（15～158）	0.985
HGB［例（％）］				
≤100 g/L	430（56）	376（61）	54（36）	
>100 g/L	334（44）	237（39）	97（64）	
APTT［s, *M*（范围）］		26.5（15.8～44.1）	26.2（17.6～40.5）	0.590
APTT［例（％）］				
延长<10 s	62（94）	47（94）	15（94）	
延长≥10 s	4（6）	3（6）	1（6）	
PT［s, *M*（范围）］		13.6（0.8～55.2）	15.7（11.3～27.6）	<0.001
PT［例（％）］				
延长<3 s	221（73）	164（79）	57（60）	
延长≥3～5.9 s	65（21）	34（16）	31（32）	
延长≥6 s	19（6）	11（5）	8（8）	
D-二聚体［mg/L, *M*（范围）］		16.5（0.34～896.9）	19.8（1.19～6593）	0.082
D-二聚体［例（％）］				
<5 mg/L	106（17）	91（18）	15（13）	
5～8.99 mg/L	70（12）	62（13）	8（6）	
≥9 mg/L	439（71）	342（69）	97（81）	
Fib［g/L, *M*（范围）］		1.3（0.24～54.8）	1.0（0.19～4.17）	<0.001
Fib［例（％）］				
<1 g/L	242（37）	179（34）	63（48）	
≥1 g/L	412（63）	345（66）	67（52）	
Cr［mg/L, *M*（范围）］		658（169～1931）	682（182～1165）	0.186
Cr［例（％）］				
≤14 mg/L	603（99）	484（99）	119（100）	
>14 mg/L	6（1）	6（1）	0（0）	
LDH［U/L, *M*（范围）］		245.0（83.0～2520.7）	512.0（171.0～2033.0）	<0.001
LDH［例（％）］				
≤245 U/L	236（42）	230（50）	6（6）	
>245 U/L	330（58）	227（50）	103（94）	
CD2［例（％）］				1.000
阳性	19（37）	18（38）	1（33）	
阴性	32（63）	30（62）	2（67）	
CD11b［例（％）］				0.026
阳性	102（27）	75（26）	27（39）	
阴性	270（73）	219（74）	51（61）	
CD56［例（％）］				0.275
阳性	89（24）	68（23）	21（28）	
阴性	283（76）	230（77）	53（72）	
PML-RARα亚型［例（％）］				0.557
L型	265（58）	223（61）	42（47）	
V型	43（9）	36（10）	7（8）	
S型	149（33）	108（29）	41（45）	
FLT3-ITD［例（％）］				0.785
阳性	111（26）	62（19）	49（52）	
阴性	308（74）	262（81）	46（48）	
FLT3-TKD［例（％）］				1.000
阳性	68（18）	46（15）	22（32）	
阴性	304（82）	257（85）	47（68）	
WT1［例（％）］				0.065
阳性	123（75）	104（87）	19（44）	
阴性	40（25）	16（13）	24（56）	
TP53缺失［例（％）］				1.000
阳性	8（8）	6（8）	2（7）	
阴性	90（92）	65（92）	25（93）	

注：Fib：纤维蛋白原；Cr：肌酐；LDH：乳酸脱氢酶。表中除WBC、PLT外，其他指标数据均有缺失

2. 细胞遗传学特征：对715例初诊APL患者进行核型分析，其中93例（13.0％）为正常核型，443例（62.0％）仅有t（15;17），155例（21.7％）为t（15;17）伴ACA，24例（3.3％）为其他染色体数目和结构异常。如图1所示，t（15;17）伴ACA中最常见的染色体异常为+8（5.5％），其次为der（15）t（15;17）（q22;q12）；而其中染色体数目异常以染色体丢失常见。复杂核型见于64例（9.0％）的患者。

**图1:**
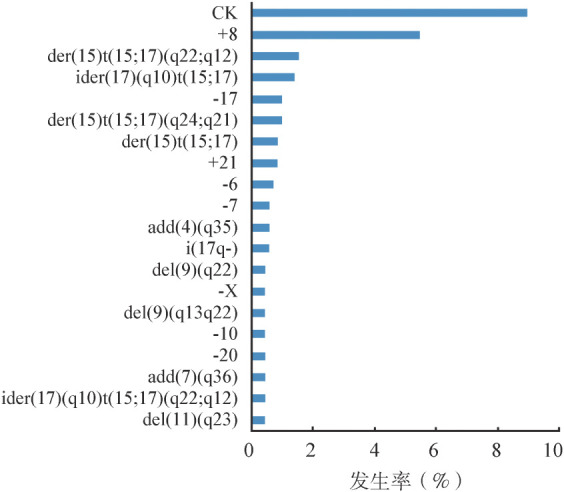
急性早幼粒细胞白血病患者t（15;17）伴附加染色体异常中出现≥3次的染色体异常

3.突变基因：采用二代测序方法对178例患者进行基因突变检测，共检测出113个突变基因。发生率>1％的突变基因有75个。如图2所示，FLT3（44.9％）为最常见的突变基因，其中FLT3-ITD见于20.8％的患者；其他较为常见的突变基因依次为WT1（21.3％）、KMT2D（14.6％）、FAT1（14.0％）、NRAS（12.4％）。

**图2:**
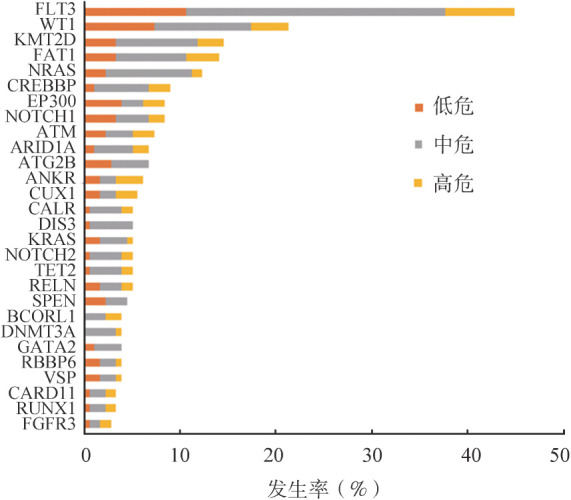
急性早幼粒细胞白血病患者突变发生率>3％的基因

## 讨论

本研究对我院15岁及以上初诊APL患者的临床及实验室特征、细胞遗传学、分子生物学情况进行了总结。初诊APL高危组患者的占比各地区报道存在差异。在一项对欧洲828例初诊APL患者（年龄<75岁）的研究中，高危患者占28.17％[11]。在一项对北美481例初诊APL患者（年龄≥15岁）的研究中，高危组患者占23.5％[12]。我院高危组患者占20％，与先前报道相似。高BMI被认为是APL不良预后的独立预测因素[13]。我院患者按照亚洲体重分层标准，超重患者比例（68％）与意大利[14]（62.5％）和北美[13]（77％）初诊患者相似；肥胖患者比例（48％）与北美[13]（50％）初诊患者相似。研究发现合并银屑病是与APL患者复发相关的独立危险因素[15]。本研究中4.8％的患者发病时伴银屑病，其在中低危组和高危组患者中的占比未见差异。已报道初诊APL中银屑病的发生率为8.3％[16]，高于普通人群中1％～3％的银屑病发生率[16]。APL典型免疫表型为CD13^+^CD33^+^HLA-DR^−^CD34^−^，伴淋系（CD2、CD56）表达的患者预后不良[17]。本研究中CD56阳性患者比例为24％，CD56阳性患者比例在中低危组和高危组中未见差异，但该比例高于Testa等[18]的报道（约10％）。

PML-RARα典型亚型包括L型、V型和S型。本研究中PML-RARα以L型最常见，比例为58％，与欧美患者相似[19]。PML-RARα亚型在不同危险组中的分布存在差异。高危组患者L型和S型比例相似，中低危组以L型为主，与先前报道不一致[20]。先前研究发现高危组以S型为主，而中低危组L型和S型比例相似。对于PML-RARα S型的预后价值尚存争议。一些研究发现伴PML-RARα S型的患者预后差，具有侵袭性病程[21]。而另外有研究显示伴S型患者的预后好或S型不影响APL患者的预后[22]。欧洲白血病网最新建议并未将PML-RARα S型列入预后因素中[21]。

本研究发现的APL突变谱与先前的报道相似，如FLT3、WT1、NRAS、KRAS和ARID1A基因的重现性改变，及其他AML罕见的突变基因（DNMT3A、TET2、ASXL1和IDH1/2）[1],[23]。FLT3为初诊APL患者最常见的突变基因，其次为WT1，与先前研究报道一致[1],[23]。本研究高危组中52％的患者伴FLT3-ITD突变，低于先前报道（约71％）[1]。本研究中KMT2D突变检出率为14.6％，其可能通过以下机制促进白血病发生：KMT2D上调抗氧化反应的基因表达程序，以防止活性氧和DNA损伤，对于MLL-AF9 诱导的白血病发生必不可少；KMT2D激活了促肿瘤的HOXA9靶基因，并且KMT2D是HOXA9和MEIS1在体内共表达介导的白血病发生所必需的[24]。FAT1最初从人类T-ALL细胞系中克隆出来；然而，FAT1很少在正常造血系统中表达。其表达见于60％的T-ALL、30％的前体B细胞ALL和10％的AML。FAT1 mRNA高表达与T-ALL患者的不良预后和高复发率相关[25]。

APL的发生除t（15;17）外还需要其他细胞遗传学改变的参与[26]。在本研究中，21.7％的患者合并ACA，低于其他报道（26％～50％）[21],[27]。其中最常见的染色体改变为+8，与先前研究结果一致[1],[21]。8号染色体额外增加的部分会导致APL细胞中MYC的下调，促进疾病发生[21]。目前研究普遍认为除复杂核型外，其他ACA并不影响患者的预后[21],[26]–[27]。伴复杂核型的APL患者复发风险增加[28]。

本中心APL的发病年龄以20～59岁为主，男女比例未见明显差异。危险分层以中低危患者为主。21.7％的患者为t（15;17）伴ACA，其中以+8最常见；9.0％的患者为复杂核型。PML-RARα 亚型以L型最常见。APL突变谱以FLT3最常见。
